# TRANSPATH^®^—A High Quality Database Focused on Signal Transduction

**DOI:** 10.1002/cfg.386

**Published:** 2004-03

**Authors:** Claudia Choi, Mathias Krull, Alexander Kel, Olga Kel-Margoulis, Susanne Pistor, Anatolij Potapov, Nico Voss, Edgar Wingender

**Affiliations:** 1 BIOBASE GmbH Halchtersche Strasse 33 Wolfenbüttel 38304 Germany; 2 Department of Bioinformatics UKG University of Göttingen Goldschmidtstrasse 1 Göttingen 37077 Germany

## Abstract

TRANSPATH^®^ can either be used as an encyclopedia, for both specific and general information on signal transduction, or can serve as a network analyser. Therefore, three modules have been created: the first one is the data, which have been manually extracted, mostly from the primary literature; the second is PathwayBuilder^™^, which provides several different types of network visualization and hence faciliates understanding; the third is ArrayAnalyzer^™^, which is particularly suited to gene expression array interpretation, and is able to identify key molecules within signalling networks (potential drug targets). These key molecules could be responsible for the coordinated regulation of downstream events. Manual data extraction focuses on direct reactions between signalling molecules and the experimental evidence for them, including species of genes/proteins used in individual experiments, experimental systems, materials and methods. This combination of materials and methods is used in TRANSPATH^®^ to assign a quality value to each experimentally proven reaction, which reflects the probability that this reaction would happen under physiological conditions. Another important feature in TRANSPATH^®^ is the inclusion of transcription factor–gene relations, which are transferred from TRANSFAC^®^, a database focused on transcription regulation and transcription factors. Since interactions between molecules are mainly direct, this allows a complete and stepwise pathway reconstruction from ligands to regulated genes. More information is available at www.biobase.de/pages/products/databases.html.


**Comparative and Functional Genomics**
*Comp Funct Genom* 2004; **5**: 163–168.
Published online in Wiley InterScience (www.interscience.wiley.com). **DOI:** 10.1002/cfg.386

**Conference Review**

### TRANSPATH® — a high quality database focused on signal transduction

Claudia Choi[1]*, Mathias Krull[1], Alexander Kel[1], Olga Kel-Margoulis[1], Susanne Pistor[1], Anatolij Potapov[1], Nico Voss[1] and Edgar Wingender[1,2]
[1] *BIOBASE GmbH, Halchtersche Strasse 33, 38304 Wolfenbüttel, Germany*
[2] *Department of Bioinformatics, UKG, University of Göttingen, Goldschmidtstrasse 1, 37077 Göttingen, Germany*

**Correspondence to:*
*Claudia Choi, BIOBASE GmbH, Halchtersche Strasse 33, 38304 Wolfenbüttel, Germany.*
*E-mail: cch@biobase.de*

Received: 7 November 2003
Revised: 15 December 2003
Accepted: 23 December 2003

### Abstract

**TRANSPATH® can either be used as an encyclopedia, for both specific and general information on signal transduction, or can serve as a network analyser. Therefore, three modules have been created: the first one is the data, which have been manually extracted, mostly from the primary literature; the second is PathwayBuilder™, which provides several different types of network visualization and hence faciliates understanding; the third is ArrayAnalyzer™, which is particularly suited to gene expression array interpretation, and is able to identify key molecules within signalling networks (potential drug targets). These key molecules could be responsible for the coordinated regulation of downstream events. Manual data extraction focuses on direct reactions between signalling molecules and the experimental evidence for them, including species of genes/proteins used in individual experiments, experimental systems, materials and methods. This combination of materials and methods is used in TRANSPATH® to assign a quality value to each experimentally proven reaction, which reflects the probability that this reaction would happen under physiological conditions. Another important feature in TRANSPATH® is the inclusion of transcription factor–gene relations, which are transferred from TRANSFAC®, a database focused on transcription regulation and transcription factors. Since interactions between molecules are mainly direct, this allows a complete and stepwise pathway reconstruction from ligands to regulated genes. More information is available at www.biobase.de/pages/products/databases.html. Copyright © 2004 John Wiley & Sons, Ltd.**

**Keywords: signal transduction; database; quality assessment; TRANSPATH; protein interaction; experimental evidence; network analysis; gene expression array**

### Introduction

Uncovering cellular processes under normal conditions will improve our understanding of pathological situations. Key components of the cellular network are potential drug target candidates and better knowledge about cellular processes will help us to learn more about possible side-effects. However, cellular networks are highly connected and complex, thus understanding them is a challenging task.

Several distinct approaches are currently used to identify molecular interactions within the cell. The classical method used by most molecular biology laboratories in the past was the investigation of single steps within the network. Generally these data are of high quality, since controls have been conducted for individual interaction results. However, this approach is not suited to the generation of holistic knowledge of cellular processes. More recently, high-throughput experiments have been developed that produce large masses of data, the most widely used methodologies being the yeast two-hybrid method [5,15], 2D-gel protein assays [4] and gene expression arrays

Copyright © 2004 John Wiley & Sons, Ltd.

[2]. Storage of these data in appropriate repositories is an absolute prerequisite for making the best use of existing research results to achieve systematic computational network analysis and to interpret these data with regard to their biological meaning. One approach for creating such databases that has been investigated is automatic text mining [12]. So far, this approach seems to be limited by several problems: e.g. protein identification is ambiguous because of the lack of a generally used terminology in the past; also, research language is heterogeneous and negations are not always interpreted correctly. Moreover, the relevant information may be extremely fragmented within individual, or even between several, publications. However, automatic text mining may provide mass information, albeit with lower quality and depth than data extraction by expert biologists. With the aim of achieving more reliable data, several interaction databases have been initiated, such as DIP [18], BIND [1] and aMAZE [16], where interactions have been inferred mostly by high-throughput experiments and mainly on yeast species. Databases concentrating more on human or mammalian proteins are CSNDB [14], TRANSPATH® [6,8,13] and MINT [19]. Among these, TRANSPATH® seems to contain the highest number of mammalian data points, in relative terms as well as in absolute numbers: over 72% of all so-called 'basic' molecules are mammalian proteins, of which about 2400 are human molecules (see Figure 1). In contrast to most of the databases mentioned above, most reactions in TRANSPATH® are directed, and hence allow upstream and downstream queries, as well as describing the function of the reaction (activation vs. inhibition). Furthermore, direct interactions between molecules are described, allowing the construction of stepwise and coherent pathways. TRANSPATH® also includes quality criteria, which are outlined below.

It is clear that we can benefit from integrating all of the major databases; their combined strength might consist in different depths of detail, simply more data on protein interactions (better coverage) or other aspects. The integration of TRANSFAC® [10] and TRANSPATH® is a good example for yielding benefits (see below). In the end more complete and broad cellular pathway knowledge will be gained.

Currently a public demo version of TRANSPATH® is available at **www.biobase.de/pages/products/transpath.html** comprising the pathway

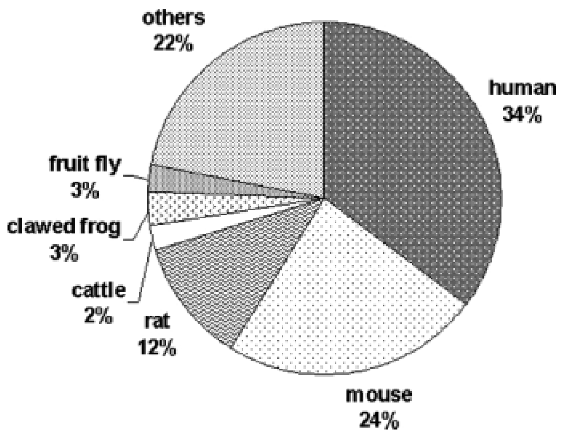

**Figure 1.** TRANSPATH® concentrates on mammalian data. Release 4.3 (October 2003) contains about 2400 human proteins, 34% of all basic proteins in the database; 70% of the basic proteins are either human, mouse or rat of interleukin-1 (IL-1), a pro-inflammatory cytokine important in the innate immune response. This extract from TRANSPATH® professional contains >600 interactions and >800 signalling molecules, mainly from mammalian organisms (which comprise 4% and 6% of TRANSPATH® Professional 4.3, respectively).

### Database structure

We segmented signal transduction into three major functional units: signalling components called 'molecules', interactions connecting these molecules called 'reactions', and 'genes' to differentiate transcriptional regulation of genes and protein interactions [8]. They are connected by a bipartite directed graph, the nodes alternately representing molecules and reactions, genes being treated as a subset of molecules. As of October 2003, TRANSPATH® Professional release 4.3 contains about 13 800 molecules, >3800 genes and >17 000 reactions collected from about 6500 references. Updates are released quarterly. Molecule and gene entries contain, among other information, cross-references to numerous other information resources, such as TRANSFAC®, SwissProt, EMBL, LocusLink, Affymetrix chips, OMIM, InterPro and GO. For some of the reactions, links have been made to DIP and BIND (200 and 20, respectively). Data are extracted mainly from the primary literature by expert

Copyright © 2004 John Wiley & Sons, Ltd. 

biologists. During the curation process the database curator specializes in specific topics in order to produce coherent networks in the database. Finalizing one specific topic will result in so-called ‘clickable maps’, summarizing expert knowledge into one cartoon, from which molecule entries are directly accessible.

Because protein constructs used in experiments are often from different species or are not indicated in primary papers, the problem arises that complete pathways cannot be constructed for a single species unless we combine the knowledge obtained from material of different species. Therefore we introduced a hierarchical representation for molecules and distinct types of reactions. For molecule entries in TRANSPATH® the lowest level corresponds to the specific molecules of a particular species, which are referred to as ‘basic’-type molecules. The second level, named ‘orthologue’-type molecules, is represented by generic entries, to which all of the corresponding orthologous basic entries are linked. This hierarchical structure enables abstractions in order to reflect interactions from several species to a more general signalling network.

Reaction entries also exist on two main levels of detail: ‘mechanistic’ reactions, which represent the mechanism of reactions and usually connect basic molecules, and ‘semantic’ ones, which depict direction and function of the reaction occurring between ‘orthologue’ molecules and hence pool information from specific ‘mechanistic’ reactions to show the signal flow in a more general manner. That way high connectivity is guaranteed with semantic reactions and detailed information can be obtained from mechanistic reactions.

### Quality assurance

Mass data in terms of protein interactions are already available (see Introduction). The remaining challenge is to structure these data to get biologically relevant answers. In our approach we rely on high quality data.

We concentrate on manual data extraction of primary publication papers particularly including data from individual or ‘small-scale experiments’ in contrast to high-throughput data or results from ‘large-scale experiments’, which often do not depict physiological conditions [3]. Manual reading and extraction of the data also enables us to pick up detailed information, in particular experimental conditions. In order to assess the relevance of a reaction for the physiological situation and the immediacy of the interaction between the reported molecules, we developed a quality scale system. Two aspects are integrated into our quality scale system: the material that has been used and the method that has been applied in a reported experiment to prove a certain reaction. For each combination of material and method we have set a ‘reliability value’ to assign a confidence level to each reaction (see Figure 2a).

During the validation process for these reliability values, a trade-off has been made between evidence of a direct interaction and physiological conditions. Detection of direct interactions is necessary for understanding the pathway in a stepwise manner, whereas physiological conditions refer to the relevance of a proven direct interaction *in vivo*. Differences in the physiological relevance arise, e.g. by *in vitro* translated material vs. correctly folded, naturally occurring, endogenous proteins.

While extracting information from a scientific paper, the origin of molecules and the methods used for proving a reaction between molecules are stored, from which the reliability value will be automatically assigned in the ‘quality’ field. By this means network analysis can be viewed and filtered according to the reliability of the underlying experimental evidences. The assigned reliability values have been revised by molecular biologists. If the user is not in agreement with our reliability values, he/she can change the values in the menu ‘edit the quality reaction matrix’ according to his own knowledge. These values will appear in the reaction table in addition to the preset ones. Along with this, a quality assessment of the experimental system, i.e. the cell lines or tissues used, is recorded. Furthermore, reactions in TRANSPATH® are often proved independently by several papers, which also confirms the reliability of a reaction (see Figure 2b).

One other quality criterion of TRANSPATH® is that species information of the interacting proteins is retained (see Figure 1), which is not the case in other databases, such as HPRD [11], where interactions reported for distinct species are all represented as part of the human network. Previous articles often have to be consulted to retrieve the species information, or the authors have to be contacted directly. This distinction is necessary,

Copyright © 2004 John Wiley & Sons, Ltd.

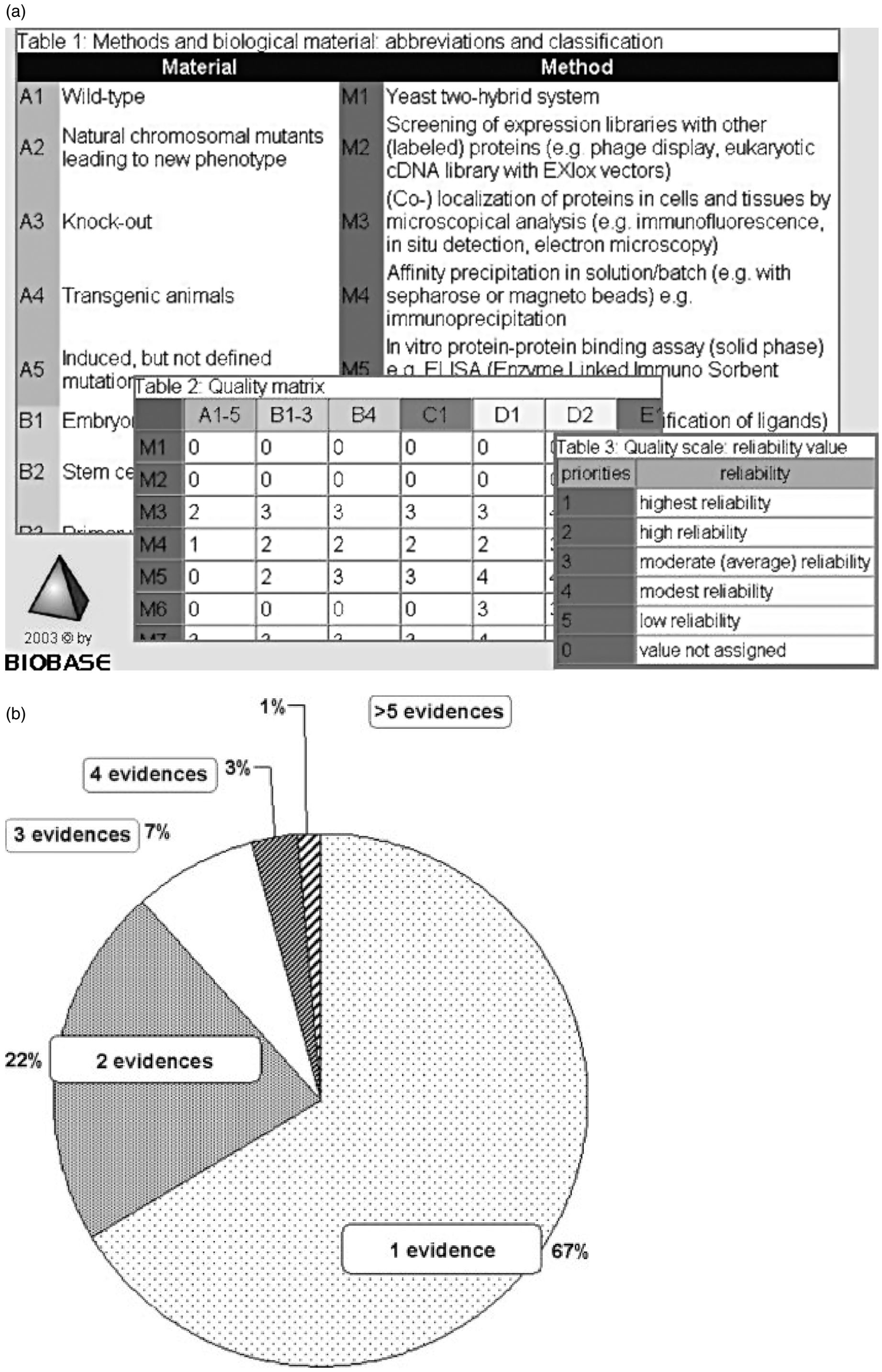

**Figure 2.** Quality assessment in TRANSPATH®. (A) Integration of data about the specific experimental conditions. Depending on the biological material and method applied in the experiment, the reaction is more or less likely to happen in the cell and organism. Therefore, we listed material sources and methods crucial for signal transduction and assigned reliability values (scale 1–5) for each combination of material and method (quality matrix). This value is provided in the reaction entry. (B) Frequency of evidences. About one-third of experimentally proven reactions are identified by more than one experiment, usually conducted using different methods by different research groups, and hence these reactions are more likely to occur *in vivo*

Copyright © 2004 John Wiley & Sons, Ltd. 

since it has been shown that orthologous proteins may exert different functions in different species [17]. Storing species details thus enables filtering in network visualization and viewing of discrepancies in signalling between different species.

TRANSPATH® is also integrated with TRANSFAC® [10], a database focused on transcriptional regulation. This allows the acquisition of high-quality data for a complete stepwise signalling pathway from ligand to gene. Hence, signalling network analysis can be directed towards transcriptional regulation or protein networks.

### Network analysis

For drug target identification it is crucial to gain knowledge on signalling networks and their key signalling components. With TRANSPATH®, target genes or key regulators can be identified. Starting with a set of genes (or their protein products) that may have been deduced from expression array data, a subnetwork can be reconstructed that comprises a maximal number of affected molecules. This can be visualized with PathwayBuilder™, in combination with ArrayAnalyzer™, which is a first step towards efficient drug target prediction. ArrayAnalyzer™ is able to find prominent molecules which are highly connected to the regulated genes. Since genes and their respective gene products (molecules) are separated in TRANSPATH®, the user can distinguish between regulation of gene expression and protein signalling networks. An example is given in Krull *et al.* [8].

### Conclusions and outlook

So far, high quality data cannot be extracted by text mining, in particular the retrieval of species and of experimental details. On the other hand, it may provide higher coverage of relevant published data and, thus, may allow us to reconstruct networks more comprehensively. Among the existing databases, only a few concentrate on mammalian species, and differentiate between gene regulation and protein networks, as in CSNDB [14], MINT [19] and TRANSPATH®. TRANSPATH® also contains tools for visualization and analysis, leading to reliable pathway and network construction.

Another point of view has been neglected so far: the spatio-temporal specificity of cellular processes. Reliable information about this is still sparse, but is expected to grow with new upcoming methods. The integration of this kind of data and their use for a more specific visualization and prediction is a key point for further development of TRANSPATH®.

Nevertheless, common efforts are desirable and will be necessary for creating a more comprehensive multi-dimensional integration, including data from all current databases in terms of protein interaction as well as signalling and metabolic pathways, from high-throughput assays, or data from text mining, and even from *in-silico* analyses, since different approaches and methodologies will strengthen the knowledge of cellular processes and thus will help to further improve our chances of combating disease.

### Acknowledgements

Many thanks to Volker Matys for proofreading. We are grateful to the whole BIOBASE team for stimulating discussions and for their help.

### References

1. Bader GD, Betel D, Hogue CWV. 2003. BIND: the Biomolecular Interaction Network Database. *Nucleic Acids Res* **31**: 248–250.
2. Dazard JE, Gal H, Amariglio N, *et al.* 2003. Genome-wide comparison of human keratinocyte and squamous cell carcinoma responses to UVB irradiation: implications for skin and epithelial cancer. *Oncogene* **22**: 2993–3006.
3. Deane CM, Salwinski L, Xenarios I, Eisenberg D. 2002. Protein interactions: two methods for assessment of the reliability of high throughput observations. *Mol Cell Proteom* **1**: 349–356.
4. Gavin AC, Bosche M, Krause R, *et al.* 2002. Functional organization of the yeast proteome by systematic analysis of protein complexes. *Nature* **415**: 141–147.
5. Giot L, Bader JS, Brouwer C, *et al.* 2003. A protein interaction map of *Drosophila melanogaster*. *Science* **302**: 1727–1736.
6. Heinemeyer T, Chen X, Karas H, *et al.* 1999. Expanding the TRANSFAC database towards an expert system of regulatory molecular mechanisms. *Nucleic Acids Res* **27**: 318–322.
7. Kel-Margoulis OV, Kel AE, Reuter I, Deineko IV, Wingender E. 2002. TransCOMPEL®: a database on composite regulatory elements in eukaryotic genes. *Nucleic Acids Res* **30**: 332–334.
8. Krull M, Voss N, Choi C, *et al.* 2003. TRANSPATH®: an integrated database on signal transduction and a tool for array analysis. *Nucleic Acids Res* **31**: 97–100.

Copyright © 2004 John Wiley & Sons, Ltd.

9. Liebich I, Bode J, Frisch M, Wingender E. 2002S/MART db: a database on scaffold/matrix attached regions.. *Nucleic Acids Res* **30**: 372–374.
10. Matys V, Fricke E, Geffers R, *et al.* 2003. TRANSFAC®: transcriptional regulation, from patterns to profiles. *Nucleic Acids Res* **31**: 374–378.
11. Peri S, Navarro JD, Amanchy R, *et al.* 2003. Development of human protein reference database as an initial platform for approaching systems biology in humans. *Genome Res* **13**: 2363–2371.
12. Rzhetsky A, Koike T, Kalachikov S, *et al.* 2000. A knowledge model for analysis and simulation of regulatory networks. *Bioinformatics* **16**: 1120–1128.
13. Schacherer F, Choi C, Götze U, *et al.* 2001. The TRANSPATH signal transduction database: a knowledge base on signal transduction networks. *Bioinformatics* **17**: 1053–1057.
14. Takai-Igarashi T, Kaminuma T. 1999. A pathway finding system for the cell signalling networks database. *In Silico Biol* **1**: 129–146.
15. Uetz P, Giot L, Cagney G, *et al.* 2000. A comprehensive analysis of protein–protein interactions in *Saccharomyces cerevisiae*. *Nature* **403**: 623–627.
16. van Helden J, Naim A, Lemer C, *et al.* 2001. From molecular activities and processes to biological function. *Briefings Bioinf* **2**: 91–93.
17. Wadhwa R, Sugihara T, Hasan MK, *et al.* 2002. A major functional difference between the mouse and human ARF tumor suppressor proteins. *J Biol Chem* **277**: 36 665–36 670.
18. Xenarios I, Salwinski L, Duan XJ, *et al.* 2002. DIP: The Database of Interacting Proteins. A research tool for studying cellular networks of protein interactions. *Nucleic Acids Res* **30**: 303–305.
19. Zanzoni A, Montecchi-Palazzi L, Quondam M, *et al.* 2002. MINT: a Molecular INTeraction database. *FEBS Lett* **513**: 135–140.

Copyright © 2004 John Wiley & Sons, Ltd.
